# Changes in adiponectin:leptin ratio among older adults with obesity following a 12-month exercise and diet intervention

**DOI:** 10.1038/s41387-022-00207-1

**Published:** 2022-06-02

**Authors:** Katelyn E. Senkus, Kristi M. Crowe-White, Anneliese C. Bolland, Julie L. Locher, Jamy D. Ard

**Affiliations:** 1 Department of Human Nutrition, The University of Alabama, Tuscaloosa, AL USA; 2 College of Communication and Information Sciences, Communication Studies & Institute for Communication and Information Research, The University of Alabama, Tuscaloosa, AL USA; 3 Division of Gerontology, Geriatrics and Palliative Care, Department of Medicine, University of Alabama at Birmingham, Birmingham, AL USA; 4 Wake Forest University School of Medicine, Department of Epidemiology and Prevention, Winston-Salem, NC USA

**Keywords:** Risk factors, Translational research

## Abstract

**Background:**

Excess adiposity is characterized by alterations in adipokine secretion such that circulating leptin concentrations are increased with reductions in adiponectin. An emerging biomarker for the assessment of this adipose tissue (AT) dysfunction is the adiponectin:leptin (AL) ratio. A low AL ratio may be suggestive of dysfunctional AT and, consequently, a heightened cardiometabolic disease risk. This ancillary study investigated the relationship between the AL ratio and cardiometabolic health among community-dwelling older adults with obesity, as well as the effects of a 12-month exercise and diet intervention on changes in the AL ratio.

**Methods:**

Participants (*n* = 163, 70.2 ± 4.7 years, 38.0% male) were randomized to the exercise only group, exercise + nutrient-dense weight maintenance group (exercise + weight maintenance), or exercise + nutrient-dense caloric restriction of 500 kcal/d group (exercise + intentional weight loss) (clinicaltrials.gov #NCT00955903). Total and regional adiposity as determined by magnetic resonance imaging (MRI) and dual-energy X-ray absorptiometry (DXA), anthropometrics, and cardiometabolic biomarkers were assessed at baseline and 12 months.

**Results:**

The AL ratio was significantly (*p* < 0.05) inversely correlated with body mass index, waist circumference, measures of adiposity, and insulin among all participants at baseline. Among females only, significant positive and inverse correlations were also observed between this ratio and high-density lipoprotein cholesterol and the inflammatory biomarkers high sensitivity C-reactive protein and interleukin-6, respectively. While controlling for biological sex, a significant time by intervention group interaction effect (*p* < 0.05) was observed such that the AL ratio significantly increased from baseline to study completion among participants in the exercise + weight maintenance group and exercise + intentional weight loss group. Post hoc analysis revealed that the exercise + intentional weight loss group exhibited a significantly greater AL ratio at study completion compared to other groups (*p* < 0.05 all).

**Conclusions:**

Results are in support of the AL ratio as a measure of AT dysfunction among older adults. Furthermore, results suggest that a 12-month exercise and diet intervention with intentional weight loss assists in improving the AL ratio in this population.

## Introduction

Obesity, a multifactorial disease characterized by excess adiposity, has been associated with increased cardiometabolic disease risk [1, 2]. This connection stems in part from the endocrine function of adipose tissue such that adipocyte-derived secretory factors, known as adipokines, influence systemic health [3]. Among several identified adipokines, leptin and adiponectin have garnered much attention due to the prominent role each plays in maintaining metabolic homeostasis. Leptin regulates food intake as well as energy expenditure, and it can also stimulate the production of pro-inflammatory cytokines [4, 5]. In contrast, adiponectin exhibits anti-inflammatory and anti-atherogenic activity with insulin-sensitizing properties [5–7].

In obesity, adipose tissue undergoes accelerated cellular and structural remodeling to compensate for excessive energy intake [8]. These adaptations detrimentally impact adipokine secretion such that circulating leptin increases with a concomitant reduction in adiponectin. The dysregulated expression of these adipokines results in adipose tissue dysfunction and, consequently, a disruption in metabolic homeostasis. With such alterations, cardiometabolic consequences are likely to ensue, including low-grade inflammation, insulin resistance, and dyslipidemia, among others [8, 9].

An emerging biomarker for the assessment of adipose tissue dysfunction is the adiponectin:leptin (AL) ratio. Previous research suggests that compared to independent measures of these adipokines, this ratio more strongly correlates with inflammation, insulin resistance, and oxidative stress, as well as a number of cardiometabolic risk factors [10–14]. As such, the AL ratio may be a practical measure for characterizing adipose tissue dysfunction and identifying individuals who are at an increased risk for cardiometabolic disease. Potential strategies to increase the AL ratio include weight loss, physical activity, and modifications to diet composition as these are related to an increase in adiponectin levels with a reduction in leptin levels [15].

Although research has demonstrated a strong relationship between AL ratio and adipose tissue dysfunction in adults, investigation of this relationship in an older adult population remains scarce. Furthermore, there is a paucity of intervention research evaluating the sensitivity of the AL ratio to lifestyle interventions. Considering this gap in the research, this ancillary study aimed to investigate the relationship between the AL ratio and cardiometabolic health among community-dwelling older adults with obesity and to assess the changes in AL ratio following a 12-month exercise and diet intervention. It was hypothesized that the AL ratio would be inversely related to adiposity and cardiometabolic biomarkers, including measures of insulin and glucose dynamics, lipids, and inflammation. Further, it was also hypothesized that an intervention combining exercise, diet quality improvements, and weight loss would result in the greatest improvement of AL ratio compared to similar lifestyle interventions without a weight loss component.

## Materials and methods

### Participants

This study was ancillary to a randomized controlled trial investigating the effects of a 12-month exercise and diet intervention on body composition and functional status among community-dwelling adults 65 years and older with obesity (Calorie Restriction in Overweight SeniorS: Response of Older Adults to a Dieting Study, CROSSROADS study; ClinicalTrial.gov #NCT00955903) [16]. By study design, participants were at risk for cardiometabolic disease with a body mass index (BMI) of 30–40 kg/m^2^ and taking at least one medication to control lipids, blood pressure, or blood glucose. Participants were excluded if they had medical, physical, or psychiatric limitations that would prevent intervention adoption and/or confound lifestyle-related body weight changes. All protocols were approved by the Institutional Review Board at The University of Alabama at Birmingham and The University of Alabama, and written informed consent was obtained from all participants for the parent study and this ancillary analysis.

### Study design

Following stratification by age (65–74, 75+ years), biological sex, and race, participants were assigned to one of the three intervention groups using a block randomization scheme: 1. Exercise only, 2. Exercise + nutrient-dense weight maintenance (exercise + weight maintenance), or 3. Exercise + nutrient-dense caloric restriction of 500 kcal/d (exercise + weight loss). To minimize potential bias, study personnel involved in data collection were blinded to group assignment.

All participants followed a standard exercise program comprised of aerobic and resistance training with home-based and gym-based activities. An exercise scientist and trainer provided tailored recommendations to participants to support weekly aerobic and resistance training goals of 90–150 min of moderate-vigorous cardiovascular exercise and two resistance training sessions, respectively. While participants were not limited to specific cardiovascular exercises, a list of common exercises was provided and a discussion of new movements to incorporate took place during group counseling sessions. Resistance training exercises were completed using resistance bands and targeted major upper and lower extremity muscle groups. Participants were given heart rate monitors to monitor the integrity of exercise sessions. One of two resistance training sessions per week was overseen by a study team member and the remaining exercise was monitored using physical activity diaries and accelerometry.

Participants in the weight maintenance and weight loss groups received counseling by a registered dietitian nutritionist (RDN) to improve dietary quality with recommendations grounded in the time-calorie displacement theory [17]. Counseling sessions were facilitated by the RDN weekly for the first six months and then every other week until study completion. Briefly, low-energy-dense fruits, vegetables, lean protein, and whole grains were encouraged with a target macronutrient intake of 25% calories from protein, 47% calories from carbohydrates, and 28% calories from fat. Both the weight maintenance and weight loss groups were given daily calorie goals based on estimates of total energy expenditure (TEE) obtained from the measured resting metabolic rate at baseline. Notably, daily calorie goals for participants in the weight loss group were 500 kcal/d less than the baseline TEE. The prescribed caloric restriction was consistent with safe weight loss recommendations for older adults outlined by the American Society for Nutrition and The Obesity Society [18]. Adherence to dietary recommendations was assessed by three unannounced 24-h dietary recalls using a multi-pass interview approach. The nutrition data system for research (NDSR), a software program validated for use among older adults, was used to generate dietary intake data (Nutrition Coordinating Center, Minneapolis, MN, 2013) [19].

### Outcome measures

Fasting blood samples were obtained at baseline and 12 months, and the following cardiometabolic outcome measures were included in this ancillary analysis; insulin, glucose, lipids, and high sensitivity c-reactive protein (hsCRP) (turbidimetric assays using reagents from Pointe Scientific, Canton, MI and a SIRRIS analyzer, Stanbio Laboratory, Boerne, TX), tumor necrosis factor-alpha (TNF-α), and interleukin-6 (IL-6) (electrochemiluminescence, Meso Scale Discovery, Rockville, MD), as well as adiponectin and leptin (radioimmunoassay and RIA reagents, respectively, Millipore, St. Charles, MO). The AL ratio was determined using the following equation: (adiponectin (μg/mL)/leptin (ng/mL) [15].

Abdominal adipose tissue (intra-abdominal, subcutaneous, and total abdominal volume) was measured by magnetic resonance imaging using a 3-Tesla Phillips Achieva System (Philips, Andover, MA, USA) at the aforementioned time points. Fat mass (total and trunk) was quantified by dual-energy X-ray absorptiometry (DXA) using a Lunar DPX-L densitometer with body composition Adult Software Version 1.33 (Lunar Corp, Madison, WI, USA). Other outcomes related to body composition included BMI and waist circumference (WC).

Blood pressure was measured using automated blood pressure devices (Omron HEM 907-XL, Omron Healthcare, Kyoto, Japan) by trained study personnel. Assessments were conducted in duplicates measured 30 s apart and averaged. In addition, a detailed methodology of the parent study has been published elsewhere [20].

### Statistical analyses

Statistical analysis was performed using SAS 9.4 (SAS Institute, Cary, NC, USA). Variables with normal distributions are expressed as mean ± standard deviation (SD), whereas, variables with non-normal distributions are expressed as median [25th percentile, 75th percentile]. Mann-Whitney U-test and Spearman’s correlations were utilized to assess the relationship between AL ratio and cardiometabolic health at baseline. A generalized linear mixed model was estimated (SASproc glimmix) using residual pseudo-likelihood to evaluate intervention effects on the AL ratio while controlling for biological sex. Given the heavily skewed right distribution of the AL ratio, it was modeled using a negative binomial error distribution with a log link function. The variance-covariance was allowed to be unstructured and parameterized through its Cholesky root. Degrees of freedom were determined using a containment method. Random effects were modeled based on *R*-side within-subject variance. Probabilities were adjusted using a method developed by Tukey and Kramer when more than two simultaneous post hoc comparisons were conducted [21]. Statistical significance was defined as *p* < 0.05.

## Results

### Study participants

**Fig. 1:**
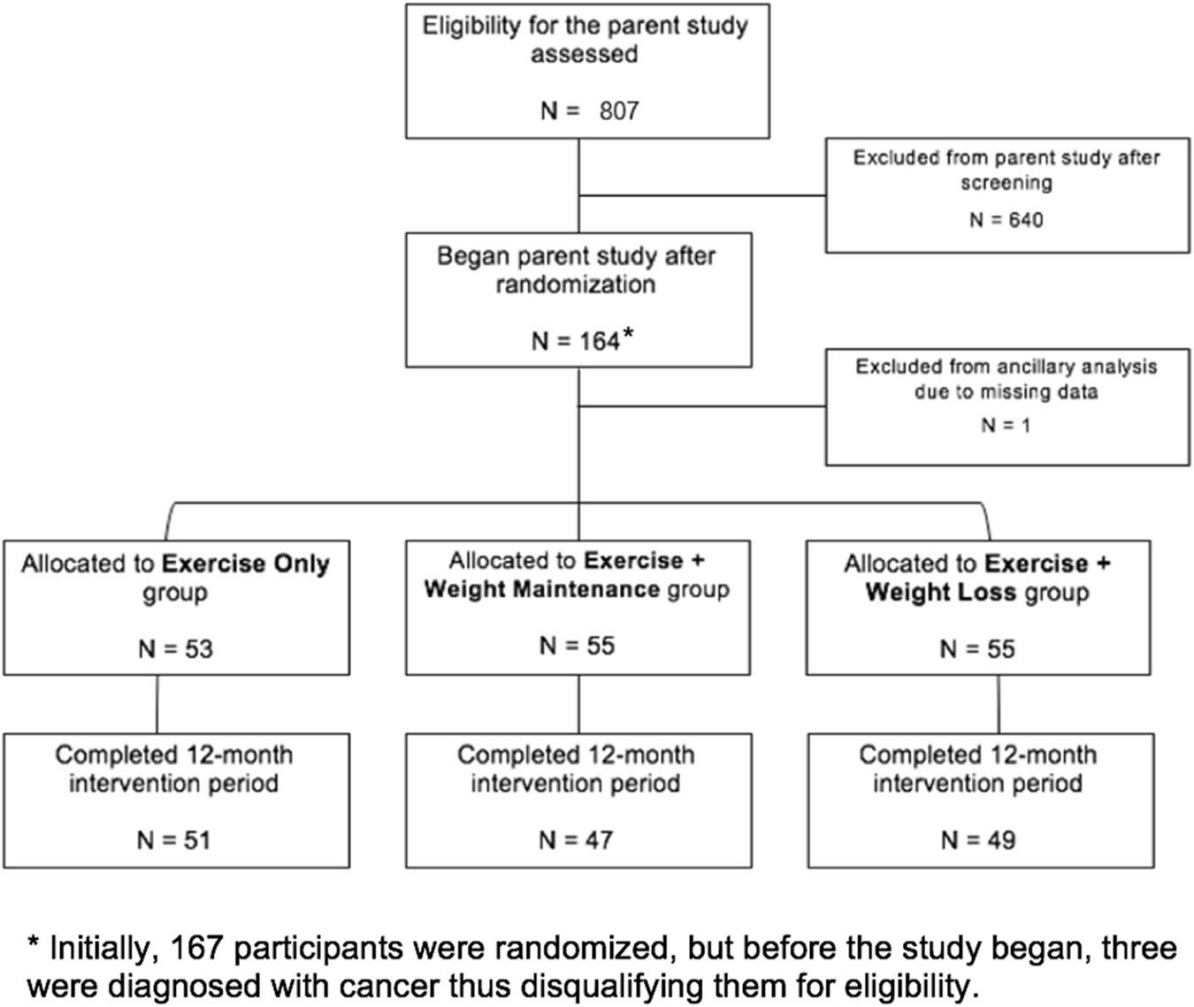
Flow Diagram for Study Inclusion. Recruitment, randomization, and allocation of a 12-month exercise and diet intervention conducted among older adults with obesity.

A total of 164 participants were enrolled in the CROSSROADS parent study. However, one participant did not have sufficient data to determine AL ratio and, thus, was excluded from this ancillary analysis (Fig. 1). Participants (*N* = 163, 70.2 ± 4.7 years, 38.0% male, 23.5% African American) were randomized to the exercise group (*n* = 53), the exercise + weight maintenance group (*n* = 55), or the exercise + weight loss group (*n* = 55). Of these, 147 participants completed the study (control: *n* = 51; weight maintenance: *n* = 47; weight loss: *n* = 49). Justification of attrition and compliance has been previously reported [16].

Participant characteristics are presented in Table 1. A significant difference in AL ratio at baseline was observed between males and females (males: 0.33 [0.21, 0.61]; females: 0.19 [0.12, 0.29], *p* < 0.001). As such, the correlational analyses were conducted separately by biological sex.

**Table 1:** Clinical and biochemical baseline characteristics among older adults with obesity following a 12-month exercise and diet intervention.

Variable	All participants (*n* = 163)	Males (*n* = 62)	Females (*n* = 101)
Age (year)	70.2 ± 4.7	70.4 ± 4.7	70.1 ± 4.6
Male sex, no. (%)	62 (38.0)	–	–
Race/ethnicity, no (%)			
European American	124 (76.1)	55 (88.71)	69 (68.32)
African American	38 (23.3)	6 (9.68)	32 (31.68)
Asian	1 (0.6)	1 (1.61)	0 (0.00)
BMI (kg/m^2^)	33.6 ± 3.0	33.7 ± 3.1	33.6 ± 3.0
WC (in)	44 ± 4	47 ± 3	42 ± 4
IAAT (cm^3^)	3478.8 ± 1808.8	4932.1 ± 1887.3	2656.8 ± 1119.3
SAAT (cm^3^)	5244.9 ± 1717.2	4710.0 ± 1616.5	2580.0 (1847.0, 3164.0)
Tot Ab volume (cm^3^)	8689.9 ± 2467.2	9557.9 ± 2747.9	8198.9 ± 2157.1
DXA total fat tissue (%)	45.8 ± 6.1	39.9 ± 4.3	49.6 ± 3.6
DXA trunk fat tissue (%)	49.8 ± 5.4	46.3 ± 4.6	52.0 ± 4.6
Insulin (μU/mL)	13.4 (10.0, 19.8)	13.8 (9.6, 20.3)	13.3 (10.2, 19.8)
Glucose (mg/dL)	103.5 (94.0, 117.0)	106.0 (96.0, 122.0)	102.5 (94.0, 115.0)
Cholesterol (mg/dL)	178.7 ± 37.7	168.4 ± 35.7	185.0 ± 37.6
Triglycerides (mg/dL)	124.5 (88.0, 158.5)	109.0 (87.5, 147.3)	132.2 ± 47.5
HDL-c (mg/dL)	54.2 ± 14.5	48.0 ± 12.1	58.1 ± 14.6
LDLc (mg/dL)	98.4 ± 32.7	95.0 ± 31.3	100.5 ± 33.6
TNF-alpha (pg/mL)	4.6 (3.8, 5.8)	4.9 (3.7, 5.9)	4.6 (3.8, 5.8)
hsCRP (mg/L)	3.0 (1.7, 6.2)	2.3 (1.2, 3.6)	4.1 (2.1, 7.5)
IL-6 (pg/mL)	2.1 (1.4, 3.2)	1.7 (1.3, 3.0)	2.2 (1.6, 3.3)
Average SBP (mmHg)	130.7 ± 13.8	133.0 ± 13.1	129.3 ± 14.1
Average DBP (mmHg)	76.0 ± 7.9	76.4 ± 8.3	75.8 ± 7.6
Adiponectin (μg/mL)	8.76 (5.92, 13.84)	6.82 (5.06, 11.64)	10.09 (6.62, 15.79)
Leptin (ng/mL)	41.68 ± 20.24	22.97 (15.94, 29.27)	52.66 ± 16.10
AL ratio	0.23 (0.15, 0.45)	0.33 (0.21, 0.61)	0.19 (0.12, 0.29)

Data are presented as mean ± SD or median (25th percentile, 75th percentile).

*BMI* body mass index, *WC* waist circumference, *IAAT* intra-abdominal adipose tissue determined by magnetic resonance imaging (MRI), *SAAT* subcutaneous abdominal adipose tissue determined by MRI, *Tot Ab volume* total abdominal fat volume determined by MRI, *DXA total fat tissue* percent total fat tissue determined by dual-energy X-ray absorptiometry (DXA), *DXA trunk fat tissue* percent trunk fat tissue determined by DXA, *HDL-c* high-density lipoprotein cholesterol, *LDL-c* low-density lipoprotein cholesterol, *TNF-α* tumor necrosis factor-alpha, *hsCRP* high sensitivity C-reactive protein, *IL-6* interleukin-6, *SBP* systolic blood pressure, *DBP* diastolic blood pressure, *AL ratio* adiponectin:leptin ratio, *N* sample size.

### Characterizing the relationship between AL ratio and measures of cardiometabolic health at baseline

Among males, significant inverse correlations between AL ratio and BMI, WC, DXA percent total fat tissue, and DXA percent trunk fat tissue were observed at baseline (*r* = −0.357, *p* = 0.004; *r* = −0.259, *p* = 0.044, *r* = −0.268, *p* = 0.035; *r* = −0.342, *p* = 0.007, respectively). Among females, AL ratio was significantly inversely correlated with BMI, WC, subcutaneous abdominal adipose tissue, and total abdominal fat volume (*r* = −0.471, *p* < 0.001; *r* = −0.385, *p* < 0.001, *r* = −0.226, *p* = 0.026; *r* = −0.293, *p* = 0.003, respectively).

For cardiometabolic biomarkers, a significant inverse correlation was only detected between AL ratio and insulin among males (*r* = −0.542, *p* < 0.001). Whereas among females, significant correlations between AL ratio and insulin, high-density lipoprotein cholesterol (HDL-c), hsCRP, and IL-6 were observed (*r* = −0.571, *p* < 0.001; *r* = 0.395, *p* < 0.001; *r* = −0.328, *p* = 0.001; *r* = −0.258, *p* = 0.010, respectively). A summary of baseline correlations is presented in Table 2.

**Table 2:** Bivariate Spearman’s correlation analyses between AL ratio and measures of adiposity and cardiometabolic health at baseline among older adults with obesity following a 12-month exercise and diet intervention.

	Males		Females	
Variable	*r*	*p*-value *n*	*r*	*p*-value *n*
BMI (kg/m^2^)	−0.357**	*p* = 0.004 *n* = 62	−0.471***	*p* < 0.001 *n* = 100
WC (in)	−0.259*	*p* = 0.044 *n* = 61	−0.385***	*p* < 0.001 *n* = 100
IAAT (cm^3^)	−0.171	*p* = 0.207 *n* = 56	−0.181	*p* = 0.074 *n* = 98
SAAT (cm^3^)	−0.062	*p* = 0.653 *n* = 55	−0.226*	*p* = 0.026 *n* = 98
Tot Ab volume (cm^3^)	−0.181	*p* = 0.181 *n* = 56	−0.293**	*p* = 0.003 *n* = 98
DXA total fat tissue (%)	−0.268*	*p* = 0.035 *n* = 62	−0.074	*p* = 0.470 *n* = 98
DXA trunk fat tissue (%)	−0.342**	*p* = 0.007 *n* = 62	−0.138	*p* = 0.176 *n* = 98
Insulin (μU/mL)	−0.542***	*p* < 0.001 *n* = 62	−0.571***	*p* < 0.001 *n* = 100
Glucose (mg/dL)	−0.076	*p* = 0.558 *n* = 62	−0.131	*p* = 0.194 *n* = 100
Triglycerides (mg/dL)	−0.178	*p* = 0.166 *n* = 62	−0.060	*p* = 0.554 *n* = 100
Cholesterol (mg/dL)	−0.059	*p* = 0.649 *n* = 62	0.072	*p* = 0.475 *n* = 100
HDL-c (mg/dL)	0.119	*p* = 0.357 *n* = 62	0.395***	*p* < 0.001 *n* = 100
LDL-c (mg/dL)	−0.087	*p* = 0.506 *n* = 61	−0.046	*p* = 0.652 *n* = 100
TNF-α (pg/mL)	−0.158	*p* = 0.220 *n* = 62	−0.171	*p* = 0.089 *n* = 100
hsCRP (mg/L)	−0.247	*p* = 0.053 *n* = 62	−0.328**	*p* = 0.001 *n* = 99
IL-6 (pg/mL)	0.069	*p* = 0.592 *n* = 62	−0.258*	*p* = 0.010 *n* = 99
Average SBP (mmHg)	−0.062	*p* = 0.634 *n* = 62	0.074	*p* = 0.464 *n* = 100
Average DBP (mmHg)	0.014	*p* = 0.915 *n* = 62	−0.041	*p* = 0.687 *n* = 100

*AL ratio* adiponectin:leptin ratio, *BMI* body mass index, *WC* waist circumference, *IAAT* intra-abdominal adipose tissue determined by magnetic resonance imaging (MRI), *SAAT* subcutaneous adipose tissue determined by MRI; *Tot Ab volume* total abdominal fat volume determined by MRI; *DXA Total fat tissue* percent total fat tissue determined by dual-energy X-ray absorptiometry (DXA), *DXA trunk fat tissue* percent trunk fat tissue determined by DXA, *HDL-c* high-density lipoprotein cholesterol, *LDL-c* low-density lipoprotein cholesterol, *TNF-α* tumor necrosis factor-alpha, *hsCRP* high sensitivity C-reactive protein, *IL-6* interleukin-6, *SBP* systolic blood pressure, *DBP* diastolic blood pressure, *r* correlation coefficient, *n* sample size.

*p < 0.05, **p < 0.01, and ***p < 0.001.

### Changes in AL ratio following a 12-month exercise and diet intervention

Generalized linear mixed model results are presented in Table 3. A significant time effect was observed such that AL ratio significantly increased among all participants from baseline to study completion (*F* (1,140 = 40.89, *p* < 0.001). There was also a significant biological sex effect (F (1,157) = 50.98, *p* < 0.001), as well as a significant intervention group by biological sex interaction effect (*F* (2,157) = 4.44, *p* = 0.013). Among the total sample and by intervention groups, males consistently exhibited a significantly higher AL ratio than females (Supplemental Table 1).

**Table 3:** AL ratio as a function of intervention group, biological sex, and time among older adults with obesity following a 12-month exercise and diet intervention.

Effect	*df*	F	*p*-value
*Main effects*			
Intervention	2,157	3.42	0.035
Time	1,140	40.89	<.0001
Biological sex	1,157	50.98	<.0001
*Interaction effects*			
Intervention × biological sex	2,157	4.44	0.013
Intervention × time	2,140	21.32	<.0001
Biological sex × time	1,140	5.84	0.017
Intervention × biological sex × time	2,140	0.58	0.563

Generalized linear mixed model results to evaluate the intervention effects on the AL ratio. Statistical significance defined as *p* < 0.05.

*AL* *ratio* adiponectin:leptin ratio.

There were no significant differences in AL ratio among intervention groups at baseline. A significant time by intervention group interaction effect was observed (*F* (2,140 = 21.32, *p* < 0.001). Analysis of simple main effects revealed that the AL ratio significantly increased from baseline to study completion among participants in the exercise + weight maintenance group (baseline adjusted mean: 0.327, 12-month adjusted mean: 0.361; *p* = 0.027) and exercise + weight loss group (baseline adjusted mean: 0.357, 12-month adjusted mean: 0.511; *p* <0.001). Improvements in AL ratio were correlated with changes in adiposity measures, notably, intra-abdominal adipose tissue levels (weight loss: *r* = −0.620, *p* < 0.001), total abdominal fat volume (weight maintenance: *r* = −0.323, *p* = 0.033; weight loss: *r* = −0.456, *p* = 0.002), and DXA percent trunk fat tissue (weight maintenance: *r* = −0.405, *p* = 0.006; weight loss: *r* = −0.423, *p* = 0.003). Despite improvements, the magnitude of changes differed such that the exercise + weight loss group exhibited a significantly greater AL ratio at study completion compared to the exercise + weight maintenance group (*p* < 0.001) and exercise only group (*p* = 0.004).

## Discussion

Obesity-associated alterations in adipokine concentrations, namely leptin and adiponectin, underpin the development of adipose tissue dysfunction, thus supporting an environment conducive to cardiometabolic disease [1, 2]. The purpose of this ancillary study was to investigate the relationship between the AL ratio, an emerging biomarker of adipose tissue dysfunction, and cardiometabolic health among older adults with obesity following a 12-month exercise and diet intervention

Previous research has consistently supported the relationship between AL ratio and cardiometabolic health among diverse populations including Korean, Japanese, Hispanic, and Slovenian adults [11, 22–25]. However, to our knowledge, this is the first study investigating the AL ratio in a biracial population of older adults. Results demonstrated that among older adults with obesity, the AL ratio was significantly associated with measures of adiposity and cardiometabolic health at baseline. In particular, correlations were observed between this ratio and BMI, WC, and measures of fat mass, as well as insulin, HDL-c, and inflammatory biomarkers. The inverse correlations related to adiposity suggest that the conventional obesity-associated alterations in adipokine secretion are captured by the AL ratio in this unique population. Similar correlations between AL ratio and body composition have been reported in Japanese adults with and without diabetes, as well as generally healthy Korean and Chinese adults [10, 22, 23, 26].

Results from the current study also support the AL ratio as a measure of adipose tissue dysfunction in the older adult population. For example, inverse correlations between this ratio and inflammatory biomarkers and insulin levels were observed, as well as a positive correlation with HDL-c. A surplus of circulating leptin nullifies its anorexigenic effects while concurrently amplifying its pro-inflammatory properties through increased activation of the nuclear factor kappa B (NFkB) signaling cascade [5, 27]. Furthermore, reductions in adiponectin inhibit signaling pathways to combat inflammation, thus perpetuating the pro-inflammatory response observed in obesity [6]. Previous research has also established a link between the AL ratio and inflammatory biomarkers, namely hsCRP and serum amyloid A (SAA), among Caucasian adults with and without metabolic syndrome [28]. Although SAA was not evaluated in the current study, this protein has been shown to increase proportionally with adiposity levels and to be responsive to weight loss interventions [29]. Accordingly, its incorporation into future investigations of the AL ratio and cardiometabolic health is warranted.

The relationship between AL ratio and insulin levels is likely driven by adiponectin. The binding of adiponectin to receptors in the liver and skeletal muscle activates signaling molecules like AMP-activated protein kinase (AMPK) and mitogen-activated protein kinase (p38 MAPK) thereby regulating glucose metabolism and insulin sensitivity [5, 7]. Zalatel et al. reported that the AL ratio was significantly associated with the euglycemic clamp-derived sensitivity index, a gold standard of insulin sensitivity assessment, and was also superior to other measures of insulin resistance, including homeostatic model assessment of insulin resistance (HOMA-IR) and the quantitative insulin sensitivity check index (QUICKI) index [11]. Strong correlations between the AL ratio and insulin levels have also been highlighted in Asian populations with and without hyperglycemia [10, 23].

Interestingly, among the lipids assessed, a significant correlation was only observed between AL ratio and HDL-c. The directionality of this relationship remains under investigation, but it is proposed that increased HDL-c enhances adiponectin secretion from adipose tissue, supporting its anti-inflammatory and insulin-sensitizing roles [30]. Nonetheless, similar relationships between the AL ratio and HDL-c have been reported [10, 14, 23]. Although previous research has not established a significant link between this ratio and LDL-c, there have been reports of an inverse correlation between AL ratio and TG levels [10, 14, 22, 23]. Such findings were not observed in the current study and may be related to a limited power to detect associations.

Acknowledging the differences in adipose tissue biology between males and females, it is suggested that biological sex be taken into account when investigating adiposity and cardiometabolic health [31]. The current study substantiates such recommendations, as males had a significantly higher AL ratio than females, irrespective of timing or intervention. Similar differences have been reported, albeit limited, and have been ascribed to inherent alterations in adipokine secretion between biological sexes [24, 25]. In general, circulating leptin levels tend to be higher among females with and without obesity compared to their male counterparts [32]. This is attributed, in part, to an increased rate of leptin secretion per unit mass of adipose tissue in females, as well as an influence of sex hormones on the release of adipokines [32, 33]. While additional mechanisms underpinning this discrepancy remain to be fully elucidated, results from the current study suggest that the AL ratio may be more sensitive to change among males.

There are a limited number of studies investigating the sensitivity of AL ratio to lifestyle interventions, and the few that exist were conducted among adolescents. For example, Masquio et al. enrolled adolescents with and without metabolic syndrome into a one-year study investigating the effects of combined nutrition and physical activity intervention on cardiometabolic health [34]. Both groups (no metabolic syndrome vs. overt metabolic syndrome) experienced an improvement in AL ratio that was associated with increases in omega-3 fatty acid consumption. Ferreira et al. implemented a non-intensive interdisciplinary therapy consisting of nutrition and exercise modifications among adolescents with obesity for 20 weeks [35]. In contrast to the first study, a significant increase in AL ratio was only observed among participants who lost >5% of body weight. While this magnitude of weight loss is generally accepted among the studied population, there is greater controversy surrounding such recommendations for older adults [36, 37]. Accordingly, the CROSSROADS parent study implemented a moderate weight loss intervention based upon recommendations outlined in a Position Statement by the American Society for Nutrition and The Obesity Society [18]. Adherence to this intervention resulted in a significant 4.1% reduction in body weight with preservation of lean body mass and bone mineral density [16]. Given the modest body weight changes observed in the current study compared to that achieved by Ferreira et al., it was unclear whether the AL ratio would be altered to a similar degree [35]. Nonetheless, the significant treatment effect observed in the current study demonstrates how each lifestyle intervention under investigation resulted in modification of the AL ratio to varying extents among older adults with obesity.

Despite overall improvements, expanded analysis of the interaction effects revealed that only participants in the exercise + weight maintenance group and exercise + weight loss group exhibited a significant increase in AL ratio from baseline to 12 months. Thus, while physical activity may influence adipokine secretion, results suggest that its benefits are amplified when combined with improvements in dietary quality and a caloric restriction of 500 kcal/d.

It is noteworthy that AL ratio benefits were observed in the exercise + weight maintenance group, as this underscores the importance of simple dietary modifications for the older adult population. Previous research suggests that certain nutrients, namely omega-3 fatty acids and fiber, may favorably alter the AL ratio [15, 38]. Although the purpose of this study was not to tease apart the individual dietary components associated with AL ratio changes, the results support how general improvements in dietary quality can have a meaningful impact on cardiometabolic health independent of weight loss. As such, recommendations to increase the consumption of fruit and non-starchy vegetables should not be underestimated by health care providers [16].

Though physical activity and dietary modifications may influence adipokine secretion, results from the current study suggest that an exercise + weight loss intervention is optimal for improved AL ratio in a community-dwelling older adult population with obesity. This is not unexpected, as weight loss is related to the restoration of balance in adiponectin and leptin levels, therefore supporting an increase in the AL ratio. These results, paired with the retention of lean body mass and bone mineral density, highlight the value of a comprehensive weight loss plan including a moderate caloric reduction and robust exercise program comprised of aerobic and resistance training.

### Strengths and limitations

Strengths of this study include robust outcome measures, notably the evaluation of visceral adipose tissue, as well as the rigorous design and monitoring of adherence in the parent study. This is also the first study to characterize the AL ratio as a measure of adipose tissue dysfunction and to evaluate its sensitivity to lifestyle interventions in a biracial older adult population. This study provides insightful results for future research, yet it is not exempt from some limitations. For example, this exploratory study was ancillary to a randomized controlled trial that was not specifically powered to detect the outcomes of this study, thus potentially obscuring some relationships. As such, an expanded investigation is warranted to discern potential differences in sensitivity of the AL ratio to lifestyle interventions by biological sex. Additionally, to enhance generalizability, it would be advantageous to evaluate the AL ratio among a greater number of Caucasian and African American older adults from various regions of the United States.

## Conclusions

Taken collectively, correlations between AL ratio and measures of cardiometabolic health support its application as a biomarker of adipose tissue dysfunction in the older adult population. The AL ratio may be clinically useful in identifying individuals susceptible to cardiometabolic disease such that a low AL ratio is reflective of dysfunctional adipose tissue and the need for intervention prior to over disease onset. Furthermore, results suggest that a 12-month exercise and diet intervention with intentional weight loss is optimal for improving the AL ratio among community-dwelling older adults with obesity. The addition of this ratio to future studies may strengthen obesity research.

## Supplementary information

Supplemental Table 1. Adjusted AL ratio means among older adults with obesity following a 12- month exercise and diet intervention.

## Data Availability

The data that support the findings of this study are available from the corresponding author, KMCW, upon reasonable request.
