# Supplementary material for: Changes in adiponectin:leptin ratio among older adults with obesity following a 12-month exercise and diet intervention
Source: Nutr Diabetes. 2022 Jun 2;12:30. doi: 10.1038/s41387-022-00207-1 (PMC9163185; doi:10.1038/s41387-022-00207-1)
Supplement: Supplementary file 1 — Supplemental Table 1. Adjusted AL ratio means among older adults with obesity following a 12- month exercise and diet intervention. [file 41387_2022_207_MOESM1_ESM.pdf]

Supplementary Information

Supplemental Table 1. Adjusted AL ratio means among older adults with obesity following a 12-month exercise and diet intervention.

	**AL Ratio (Adjusted Means)**	
	Males	Females
Overall	0.488	0.296
By intervention		
Exercise only	0.557	0.252
Exercise + weight maintenance	0.396	0.298
Exercise + weight loss	0.528	0.345

Generalized linear mixed model results to evaluate the intervention effects on the AL ratio. Adjusted means for the reported significant biological sex effect ($F (1, 147) = 50.98$, $p = 0.05$), as well as the significant intervention group by biological sex interaction ($F (2, 157) = 4.44$, $p = 0.013$), simple main effects analysis. Using least squares, means were adjusted for other main effects and interactions. Statistical significance defined as $p < 0.05$. AL ratio = adiponectin:leptin ratio; exercise plus weight maintenance = exercise + nutrient-dense weight maintenance diet intervention; exercise plus weight loss = exercise + nutrient-dense caloric restriction of 500 kcal/day intervention.
